# The Hospitals Again Vindicated!

**Published:** 1889-11-09

**Authors:** 


### The Hospitals Again Vindicated!

Once again the management of our hospitals has been vindicated, and a false charge refuted. Mr. Conrad W. Thies, the secretary of the Royal Free Hospital, in a letter to the *Daily Chronicle*, which always does its best to help all the charities, writes :

"My attention has been drawn to an article in to-day's issue of your paper (Nov. 4), in which appears the following statement: A man was brought by the police to a 'well-known hospital in the Gray's-inn-road in a critical condition, was looked at by an official qualified to form an opinion on the new-comer's state, and with the result that he was pronounced to be merely drunk, and consequently ineligible for admission.' The article further states that the man was taken away by the police to the workhouse infirmary, 'where he was found to be in a dangerous condition.'

"The real facts of the case are as follows: The man referred to was brought to this hospital about half-past one a.m. on Saturday morning, suffering from slight wounds caused by a fall. He was in a drunken and excited condition, and was only kept still for his wounds to be dressed by the two policemen who had brought him in. Whilst his wounds were being attended to he was noisy and foul in language. As there was no serious injury he was, after nearly an hour's detention, taken away by the police to be charged for drunkenness and disorder in the streets. By order of the divisional surgeon he was removed to the workhouse infirmary.

"I have this morning seen the medical officer in charge of the infirmary, who informs me that in his opinion our medical officer did all that was possible under the circumstances. When brought to the infirmary the man was suffering from the effects of excitement caused by drink, and is at present detained on account of chronic bronchitis, for which he had on a previous occasion been under treatment at the infirmary.

"Under these circumstances, I trust that you will give publicity to this brief statement of the facts of the case, upon which your writer has commented, as the article is calculated to do serious injury to this institution."

